# Ultrasound-Guided Percutaneous Drainage of Iliopsoas Abscess With Septicemia in an Adolescent: A Case Report and Literature Review

**DOI:** 10.3389/fsurg.2022.871292

**Published:** 2022-06-27

**Authors:** Kun Jiang, Wenxiao Zhang, Guoyong Fu, Guanghe Cui, Xuna Li, Shousong Ren, Tingliang Fu, Lei Geng

**Affiliations:** ^1^ Department of Pediatric Surgery, Binzhou Medical Unversity Hospital, Binzhou, China; ^2^ Department of Ultrasonic Medicine, Binzhou Medical Unversity Hospital, Binzhou, China

**Keywords:** staphylococcus aureus, ultrasonography, percutaneous catheter drainage, pediatrics, iliopsoas abscess

## Abstract

**Introduction:**

Iliopsoas abscess with septicemia in the pediatric population is rare. Early diagnosis and effective management of this emergent disorder remain challenging for clinicians.

**Case Presentation:**

A 14-year-old girl presented with right lateral and posterior hip pain and fever for 7 days before admission. Blood culture was positive for *Staphylococcus aureus.* Enhanced magnetic resonance imaging revealed abscesses located in the right iliopsoas muscle and on the surface deep to the fascia of the right sacroiliac joint that were 6.8 cm × 6.2 cm × 5.7 cm and 3.7 cm × 3.5 cm × 2.1 cm, respectively. A diagnosis of right iliopsoas abscesses with septicemia was made. The patient received intravenous antibiotics, underwent ultrasound-guided percutaneous catheter drainage, and recovered uneventfully. Medical literature regarding this issue published in the English language during the last two decades was reviewed.

**Discussion:**

Primary synchronous psoas and iliacus muscle abscesses are rare and emergent disorders in the pediatric age group. The diagnosis is generally delayed owing to the deep anatomic location and nonspecific signs and symptoms. A comprehensive medical history, meticulous physical examination, and judicious use of imaging studies could establish a timely and accurate diagnosis. Surgeons should be aware of the occurrence of multiple abscesses. Prompt and adequate antibiotic therapy accompanied by a mini-invasive approach, such as ultrasound-guided, laparoscopic, or video-retroperitoneoscopic drainage of the infectious focus, if indicated and feasible, is important to achieve a good outcome in the management of iliopsoas abscess.

## Introduction

Synchronous iliacus and psoas abscesses with septicemia are extremely rare, particularly in children (1). The clinical presentation of suppurative iliopsoas abscess is often subacute, and its differential diagnosis includes lumbar vertebral osteomyelitis, septic sacroiliitis, and regional suppurative lymphadenitis (2). Moreover, limited hip joint motility may occur even in the absence of actual involvement of the hip. Nonspecific signs and symptoms of iliopsoas abscess may also mimic septic arthritis of the hip (3, 4). Owing to the deep location of the infection and its adjacent structures, including the intestine, iliac vessels, and femoral nerve, it is difficult to identify a safe approach for draining the abscesses percutaneously. Ultrasound-guided aspiration and catheter drainage are most commonly used for single abscess, while open surgery is recommended for complex abscess (3, 5–7). Herein, we present a case of an adolescent with iliopsoas abscess accompanying septicemia. The patient recovered uneventfully with ultrasound-guided percutaneous catheter drainage and intravenous antibiotic therapy. The literature on its pathophysiology, diagnosis, and management is also reviewed.

## Case Description

A 14-year-old female patient was brought to an outpatient clinic because of right hip pain and limp with mild fever for 7 days, for which she received acupuncture therapy and intravenous antibiotic therapy. Her pain worsened with persistent high fever, reaching 40°C. The patient had no cough, shortness of breath, vomiting, or abdominal pain or distension. There was no history of food or drug allergy or trauma, except for a history of long jump exercise 1 day before the onset of hip pain.

A physical examination demonstrated that the patient flexed her right thigh and rotated it outward along with the right sacroiliac joint, and she had paralumbar (L_4, 5_) tenderness.

The laboratory investigation showed a white blood cell (WBC) count of 14.5 × 10^9^/L, neutrophil percentage of 77.4%, neutrophil count of 11.3 × 10^9^/L, serum C-reactive protein (CRP) level of 179.10 mg/L (reference range for inflammation, <10), and erythrocyte sedimentation rate (ESR) of 120 mm/h. Plain radiographs of the pelvis showed that the articular surface was blurred with unwidened joint space. Soft tissue infection or inflammation adjacent to the right sacroiliac joint was suspected. An ultrasound examination revealed no abnormal alterations in the early stage.

**Figure 1:**
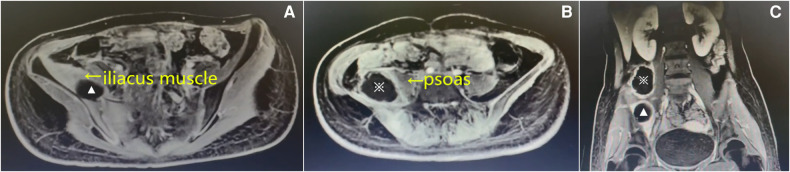
Enhanced magnetic resonance imaging (MRI) on the 3rd admission day. Axial (**A**) and (**B**) and coronal T1-weighted images (**C**) showing that the abscesses were located in the right iliacus muscle (▴) and posterolateral to the right psoas (※).

After admission, the patient received intravenous antibiotics (cefazolin sodium) at a dose of 1 g bid. After treatment for 2 days, there was a slight reduction in the fever; however, no significant improvement was noted in the regional pain. On hospital day 3, the blood cultures were positive for *Staphylococcus aureus*, which was sensitive to oxacillin. Enhanced magnetic resonance imaging (MRI) revealed an abscess located posterolateral to the right psoas and was 6.8 cm × 6.2 cm × 5.7 cm, while another one was located within the swollen right iliacus muscle and was 3.7 cm × 3.5 cm × 2.1 cm (Figure 1). The diagnosis of synchronous primary right iliacus and psoas abscesses with septicemia was confirmed. Intravenous antibiotics (cefoperazone sulbactam) were administered based on the results of blood cultures and antibiotic sensitivity tests.

**Figure 2:**
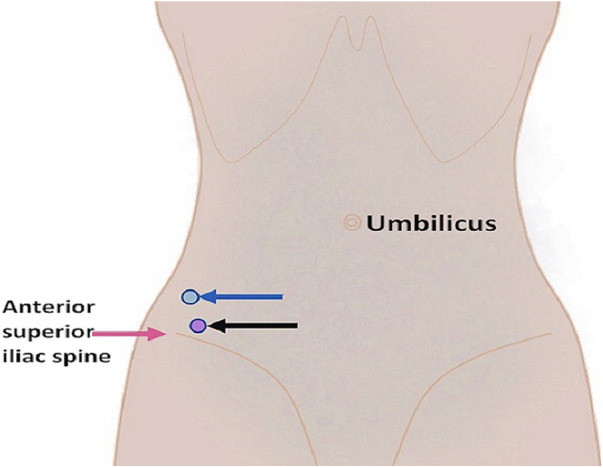
Diagram illustrating the puncture sites for the psoas abscess (blue arrow) and the iliacus muscle abscess (black arrow).

The fever and regional pain were relieved; however, the patient still could not move her right leg. Ultrasound images revealed mixed echo cystic lesions located posteriorly to the right psoas and within the swollen right iliacus muscle that were 7.1 cm × 4.3 cm and 4.9 cm × 1.9 cm, respectively. Ultrasound-guided percutaneous catheter drainage of the abscess was planned and performed on hospitalization day 14. The abscesses were drained via a different anterior route above the anterior superior iliac spine (Figure 2). Using the Seldinger technique, two 8-F pigtail drainage catheters were placed separately in the abscess cavities, and 50 mL of thick yellowish pus from the psoas abscess and 30 mL of thin yellowish pus from the iliacus muscle abscess were drained out. The catheters were then properly fixed and connected to two aseptic suction drainage systems. The growth of *S. aureus* was observed in the pus from both abscesses, and it was sensitive to oxacillin.

The intravenous administration of cefoperazone sulbactam was continued for 2 weeks. Normal saline was intermittently applied to flush the abscess cavities separately to ensure sufficient drainage. The regional pain was significantly relieved on the first day after drainage. On the third day postdrainage, the patient was afebrile and could leave the bed and move around independently. On the fifth postoperative day, the drainage of the collection was significantly reduced. Ultrasonography revealed no obvious residual abscesses on the eighth day after drainage, and the WBC count and serum CRP level were within the normal range (10 mg/L). The bacterial culture of the drainage fluid was negative. The drainage catheters were removed on the 12th postdrainage day. The patient was discharged from the hospital on the 14th postdrainage day.

In a 6-month follow-up period by a telephone interview or the outpatient clinic, the patient recovered completely.

The medical literature was scanned for pediatric iliopsoas abscess, iliopsoas abscess in children, psoas abscess in children, or psoas abscess in neonates during the last two decades (from January 2002 to December 2021). There are 44 papers published in the English language, and the data associated with these issues are usually in the form of case report(s) (8–50). Clinical data included age (0–18 years), gender, medical history, symptoms and signs, inflammatory parameters, bacterial culture, imaging, treatment options, and outcomes. Sixty-nine cases met the inclusion criteria. The clinical characteristics are summarized in Table 1. Onset time has ranged from newborn to 18 years of age, about 75% of the cases have been reported in the age group of 4–18 years, and the ratio of male to female is 1.5:1. Only 1.3% of the cases have predisposing factors, such as diabetes, immune deficiency, and suspected trauma. Fever is the most common symptom; however, 1.3% of the cases are afebrile, especially in newborn patients. More than half of the cases present hip mobility limitations and a painful hip. Our case displayed high fever, painful hip, and poor mobility with a flexion deformity. The diagnosis delay ranges from 4 to 30 days. Nearly all cases with primary iliopsoas abscess have significant elevations in inflammatory parameters, such as WBC and CRP levels. Ultrasonography and/or MRI are the main imaging tools used for making the diagnosis. In the present case, MRI indicated the abscess location. In most cases, the cause is not detected; however, 11 cases have secondary abscess from inflammation of perforated appendicitis, sacroiliitis, or Crohn’s disease with fistula. *S. aureus* is the most frequently isolated organism from blood and/or pus in pediatric iliopsoas abscesses. Five cases have methicillin-resistant *S. aureus* infection. The second causative microorganism isolated is *Streptococci*. Regarding the management of pediatric iliopsoas abscess, antibiotic therapy alone is sufficient in 11 cases; however, abscess aspiration or drainage is required in nearly 85% of the cases, including more than half of them undergoing open surgical drainage. In most cases, the duration of antibiotic therapy lasts over 3 to 6 weeks. The outcome of iliopsoas abscess usually is good.

**Table 1:** Summary of the clinical characteristics (8–50), *N* = 69.


Age (years)	
0–3	17
4–12	29
13–18	23
Gender	
M	38
F	24
unknown	7
Predisposing factors	
unknown	60
diabetes	4
immune deficiency	1
trauma	3
bone marrow aspiration	1
Medical history	
duration of symptoms (day)	
0–3	3
4–7	10
8–30	11
unknown	45
Symptoms and signs	
fever (afebrile)	60 (9)
painful hip or thigh	33
hip movement limitation	31
inguinal area pain	11
abdonimal pain	13
back pain	12
walking difficulty	12
hip flexion deformity	39
abdominal tenderness	6
Elevations of inflammatory markers	
WBC	63
CRP	65
ESR	68
Bacterial culture (positive/negative)	
blood	17/11
pus	26/3
Microorganism growth	
*S. aureus*	33
methicillin-sensitive	12
methicillin-resistant	5
*S. epidermis*	1
*Streptococci*	5
*Salmonella*	2
*E. faecalis*	2
Imaging diagnosis (confirmed/unconfirmed)	
USG	27/5
MRI	30
CT scan	12/1
Treatment options	
conservative observation	11
USG/CT-guided percutaneous drainage or aspiration	20
open surgical or retroperitoneal laparoscopic approach	38
Duration of the antibiotics (week)	
3–6	28
7–15	7
unknown	34
Concomitant	
sickle cell anemia	1
erythematous systemic lupus	1
acute appendicitis	2
sacroiliitis	3
pyogenic hip arthritis	3
Crohn’s disease	1
perirenal abscess	1
multiple vein thrombosis of the inferior vena cava	1
Outcome	
cure	67
recurrent	1
in-hospital death	1

## Discussion

Iliopsoas abscess was usually classified as being primary or secondary. Hematogenous spread of causative pathogens from a distal focus can cause primary iliopsoas abscess. The spread from an adjacent suppurative lesion, including appendicitis, Crohn’s disease, pancreatitis, pyelonephritis, ipsilateral septic arthritis, spondylodiscitis, lumbar vertebral osteomyelitis, septic sacroiliitis, and thoracolumbar spinal tuberculosis, can cause secondary iliopsoas abscess. Iiopsoas abscess in the pediatric population is rare and often primary. Although the precise cause of iliopsoas abscess is unclear, bacterial lymphadenitis, previous traumatism (as in the present case), and hematogenous seeding of the bacteria have been proposed as initial factors (51–56). Transient bacteremia can affect previously healthy children after strenuous or vigorous exercise or following localized and possibly unnoticed trauma (1, 2), as in the present case. Patients with decreased immune response, especially with primary immune deficiencies, will be more susceptible to pyogenic infections, including iliopsoas abscess. In more than three-fourths of cases, *S. aureus* has been causing bacteria in primary iliopsoas abscess, but for the secondary type, Gram-negative or anaerobic bacteria usually grow on pus culture (51–56).

In general, iliopsoas abscess has a subacute clinical course, and patients seek medical assistance in an average of 5–6 days after the onset of signs and symptoms. Children may present with a more acute presentation (3, 57). Pyomyositis, including iliopsoas abscess(es), has three consecutive clinical stages (58, 59). The onset is generally insidious with edema and pain in the involved muscle group. Only a few patients (approximately 2%) present at this early stage (diffuse muscle infection). Most patients present in the second phase (abscess formation). The third phase (sepsis) is characterized by the presence of systemic toxic symptoms such as high fever or septic shock (58, 59). The present patient had a history of extenuating exercise a day before the onset of the disorder and progressed rapidly, and the organism of blood and pus cultures was *S. aureus*, which is consistent with most cases reported in the literature.

Suppurative nontuberculous abscesses of the psoas and iliacus muscles in children are uncommon (1). The variety of symptoms and signs resembling those of primary infection in the hip or intra-abdominal organs and the deep location of the muscles make early and accurate diagnosis challenging (60). Delays in diagnosis are the major cause of morbidity and can even be life-threatening (5–7). Regional pain corresponding to the affected area, a common symptom of iliacus and psoas abscess, occurs in more than 90% of cases in the pediatric population (7). In the present case, the patient experienced hip pain after strenuous exercise 1 day before the onset of the hip pain. The pain was aggravated, and a high fever occurred. However, some cases of iliacus and psoas abscesses have no typical clinical manifestations. Acute pain in the lumbosacral region, buttocks, groin region, hip joint, and thigh should be considered for this condition (8, 54). Although blood tests are often nonspecific, the abnormal elevation of WBC counts, ESR, and CRP indicates the existence of an acute systemic inflammatory response (8). In the present case, the blood and pus cultures were positive for *S. aureus*, suggesting primary synchronous iliacus and psoas abscesses. Early blood and pus bacterial cultures are vital to assist clinicians with selecting appropriate antibiotics for the management of this acute condition (6, 61). Despite imaging investigations, a high index of suspicion of this disease and the use of imaging modalities, such as repeated ultrasonography or MRI with contrast, may help confirm the diagnosis earlier (61, 62). Ultrasonography is a common and important method for diagnosing iliopsoas abscess (55). Ultrasonography is free from radiation effects and easy to use; however, it is a practitioner-dependent diagnostic tool. Moreover, it is difficult to display the retroperitoneal area owing to the presence of intestinal gases (8). In our case, an ultrasound examination was performed, and no significant alterations were found due to the absence of abscess formation in its early stage. A negative ultrasound is insufficient to exclude iliopsoas abscess. Repeated point-of-care ultrasonography and further investigations are recommended to ensure an early diagnosis (63). MRI is the preferred diagnostic imaging modality which can play an important role in the early recognition of bacterial pyomyositis (58, 59). MRI can provide useful information to clinicians to make an early diagnosis and reveal the precise site and size of the abscess. Furthermore, MRI is preferred because it is superior to computed tomography (CT) for displaying soft tissues and does not require intravenous contrast material to display the abscess wall and peripheral structures (61, 62). In our case, an enhanced MRI was conducted, and two separate abscesses were revealed and confirmed later by ultrasound-guided percutaneous catheter drainage. We recommend that patients suspected of having iliopsoas abscess undergo MRI for early diagnosis and treatment planning.

The management of this emergency status should consist of the early use of appropriate antibiotics along with abscess drainage. Due to the deep location and adjacent structures, such as the intestine, iliac vessels, and femoral nerve, it is difficult to identify a safe approach for placing a catheter to drain the abscesses percutaneously. The common organism causing iliacus muscle or psoas abscess is *S. aureus*, and antistaphylococcal antibiotics should be administered as early as possible after admission (64, 65). The patient in the present case received intravenous cefazolin sodium after admission; thereafter, the antibiotic was adjusted to cefoperazone sulbactam according to the positive blood culture of *S. aureus*. Accordig to the patients’ clinical status and lesion size, patients generally receive antibiotics for 1 week to 10 days intravenously, for a total of 5–6 weeks orally (58). Occasionally, intravenous therapy is administered for 6 weeks because the organism is multiresistant to oral antibiotics (66). In the present case, the duration of antibiotics depended on the clinical response, and antibiotics were continued for at least 2 weeks after drainage and fever resolution.

There are a few reports regarding image-guided percutaneous drainage for the management of iliacus and psoas abscesses. Most patients could be managed using radiologically or ultrasound-guided percutaneous drainage with optimal results, even in a neonate with an iliopsoas abscess (9, 10). Ultrasound-guided drainage of the iliacus and/or psoas abscess(es) conducted by experienced physicians has several benefits, including precision, minimal invasiveness, fast recovery, avoidance of radiation exposure, and invisible scars (67). Ultrasound-guided percutaneous needle aspiration or catheter drainage might be the first choice according to the available facilities and experienced physicians in ultrasonic medicine (8, 11, 56, 68). In our case, using the ultrasound-guided method, two 8-Fr pigtail catheters were successfully placed in two separate abscesses. An intermittent flush with normal saline was recommended to ensure sufficient drainage. However, if a high risk of adjacent important structure injury related to aspiration or multiloculated abscess with very thick pus was assessed by imaging, laparoscopic-assisted, retroperitoneoscopic-assisted, and even traditional open surgical drainage (6, 10, 12, 13, 62, 69–72) remains an alternative option.

The indication for catheter removal may include the following parameters: complete regional pain relief, normal body temperature for more than 3 days, freely able to leave the bed, WBC count and CRP levels within the normal range, negative drainage fluid bacterial culture, and disappearance of the abscess cavity on ultrasonography (73).

In conclusion, primary iliopsoas abscess is extremely rare. The diagnosis is generally delayed owing to the deep location and unspecific signs and symptoms. A comprehensive medical history, meticulous physical examination, and judicious use of imaging studies could establish an accurate diagnosis. MRI is invaluable for establishing the diagnosis and anatomical site. Surgeons should be aware of the occurrence of multiple abscesses. In the era of minimally invasive surgery, prompt and adequate antibiotic therapy accompanied by mini-invasive approaches, such as ultrasound-guided, laparoscopic or video-retroperitoneoscopic drainage of the infectious focus, if indicated and feasible, is important to achieve a good outcome and significantly decrease the overall morbidity and mortality in the management of iliopsoas abscess (56, 74–76).

**Figure 3:**
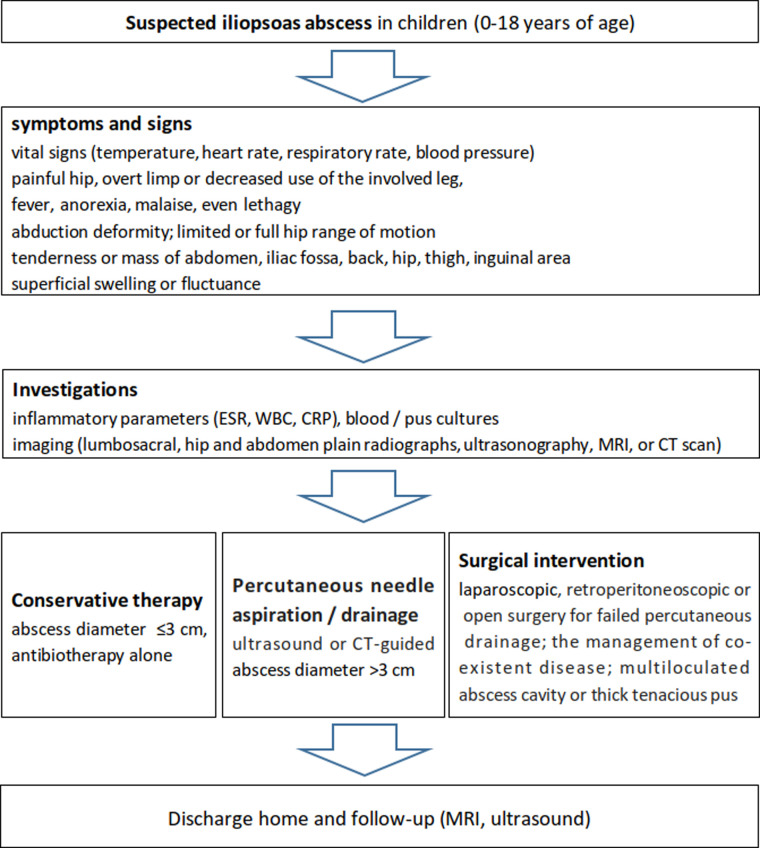
Recommended flow chart for the diagnosis and management of iliopsoas abscess in the pediatric population according to the literature and our experience.

Based on the literature about pediatric iliopsoas abscess (51, 53, 56, 12, 77–93), a flow chart of its diagnosisa and treatment was recommended as shown in Figure 3.

## Data Availability

The raw data supporting the conclusions of this article will be made available by the authors without undue reservation.
